# Discursive construction of the ‘scholar' identity: a critical genre analysis of chinese and english academic biographies

**DOI:** 10.3389/fpsyg.2025.1581772

**Published:** 2025-08-19

**Authors:** Shuai Liu

**Affiliations:** Institute of Curriculum and Instruction, Faculty of Education, East China Normal University, Shanghai, China

**Keywords:** scholar biography, CGA, discourse practices, identity construction, comparative study

## Abstract

Scholar biographies, though often overlooked, serve as a significant genre for the discursive construction of academic identity. This study adopts a Critical Genre Analysis (CGA) framework to examine how professional identities are constructed in Chinese and English scholar profiles within the field of linguistics. Combining textual analysis with interview data, the research identifies shared rhetorical structures and recurring identity types, alongside notable cross-cultural differences shaped by institutional, and socio-cultural factors. The interviews further reveal how scholars strategically present themselves in response to contextual expectations. By integrating textual and contextual perspectives, the study deepens our understanding of how academic identities are constructed and culturally mediated, offering implications for academic communication and cross-cultural discourse research.

## 1 Introduction

Identity has been widely conceptualized in contemporary scholarship as a dynamic, fluid, and contextually situated construct (Benwell and Stokoe, 2006). It is not an innate or fixed attribute, but rather an ongoing process of construction and reconstruction, shaped by multiple intersecting factors. Among these, prior educational experiences, as well as the operation of discourse, agency, and individual life narratives (Mili and Towers, 2022), play a particularly salient role. Situated at the nexus of the individual and the social, identity encapsulates the dialectical interplay between personal agency and the normative structures of society. Anchored in the theoretical framework of social constructivism, identity is not conceived as a stable core self but as a reflexively produced and discursively mediated phenomenon. It is constituted and communicated through a range of semiotic resources, including linguistic choices, discursive practices, stylistic performances, affective stances, and embodied behaviors (Burkitt, 2008). In this regard, identity is both a performative act and a socio-cultural artifact, continuously negotiated within specific communicative contexts and institutional settings.

A particularly salient genre through which academic identity is explicitly performed and negotiated is the scholar biography—a concise self-representational narrative that outlines an individual's academic trajectory, professional accomplishments, and intellectual positioning (Haiying, 2020). Commonly featured on academic websites, conference programs, editorial notes, and lecture introductions, the scholar biography operates as both a medium of self-presentation and a site where broader institutional, disciplinary, and market forces converge. It reflects not only how individuals choose to articulate their academic selves, but also how these articulations are shaped, constrained, and legitimized by prevailing socio-institutional norms and expectations.

Despite its strategic function within the academic field, the scholar biography has received limited scholarly attention and is often relegated to a peripheral position in academic discourse. This marginalization may be attributed to the genre's typically formulaic and condensed nature, which tends to constrain opportunities for extended narrative or nuanced self-positioning (Tse, 2012). Nonetheless, in an era characterized by the increasing emphasis on personal branding, digital visibility, and academic performativity, the scholar biography warrants closer critical scrutiny. It serves as a discursive site through which broader dynamics of identity construction, institutional power, and knowledge legitimation are rendered visible and contested.

## 2 Literature review

Discourse, as a central mediating resource in social interaction, plays a constitutive role in identity construction. It is not merely a conduit for information transmission, but a socially and semiotically rich medium through which individuals construct subject positions, negotiate relational dynamics, and perform multiple—and often intersecting—social roles (Cidlinska et al., 2023). Recent scholarship has emphasized that discourse operates as a dynamic arena for identity enactment, enabling individuals to mobilize linguistic and interactional resources to align with or resist prevailing norms (Dai and Hardy, 2024). In academic settings, such discursive negotiations often occur within highly regulated and genre-specific communicative spaces, where the performativity of identity is closely tied to the strategic deployment of disciplinary and institutional discourse (Guan and Xu, 2024).

The interplay between genre, discourse, and identity offers a productive analytical lens for understanding how self-representation is structured and constrained by context. As scholars have noted, genre serves as both a structuring convention and a site of rhetorical agency (Hyland, 2011; Yunling, 2023). It provides the communicative expectations within which discourse operates, while simultaneously enabling individuals to exercise choices in identity positioning. In this view, identity is not simply reflected through genre, but actively co-constructed through the situated deployment of discursive resources within genre-specific contexts.

Identity construction, then, may be understood as a process of recognition, positioning, and affiliation (Greek and Jonsmoen, 2021), moving from reflexive self-awareness to strategic social alignment. The process is inherently contextual, shaped by variables such as time, space, institutional norms, and personal biography. For example, (Yingzhe 2021) demonstrates how academic presenters engage in metapragmatic discourse to perform identity awareness and manage audience reception during conference presentations. This aligns with Guan and Xu's (2024) findings on how scholars craft self-representations in alignment with disciplinary expectations, illustrating a shared concern with the performative and strategic nature of academic identity within institutionalized genres.

Complementary insights are offered by Xinren and Mengxin (2016), who examine how academic conference moderators adapt their discursive strategies to different participant roles and interactional settings. Their study underscores the pragmatic versatility with which individuals shift between identities depending on audience and institutional positioning—a point that resonates with Hyland (2011) observation that academic self-presentation is shaped not only by professional hierarchy and gender, but also by the constraints and affordances of specific written genres, such as personal webpages.

The significance of genre in identity performance is further illuminated in Tse (2012) case study of autobiographical discourse. Analyzing Ray Kroc's memoir, Zhang identifies how narrative stance, pronoun usage, and evidentiality function as rhetorical strategies for asserting personal identity. While situated outside formal academic genres, the study offers transferable insights into how linguistic choices within genre frameworks contribute to identity projection—echoing the broader claim that identity is not expressed in isolation but always mediated by discursive and generic form. Hyland (2011) builds directly on this discourse-genre-identity nexus through his analysis of 100 academic personal webpages, showing how identity is constructed through patterned rhetorical moves shaped by gender, academic status, and institutional positioning, and therefore he proposed that academic identity involves the careful balancing of personal voice and disciplinary alignment—a process that unfolds within genre-based expectations. This insight establishes a critical dialogue with Yunling (2023) on rhetorical agency, and with Dai and Hardy (2024) on the multiplicity of voices in identity performance, collectively reinforcing the central role of genre as both an enabler and a delimiter of discursive identity construction.

Taken together, these studies demonstrate that identity is a socially situated, discursively mediated, and genre-conditioned construct. It emerges not solely from individual intention, but from the intersection of personal expression, institutional structures, and the rhetorical conventions of genre. Despite increasing scholarly attention to identity in academic discourse, the scholar biography—a condensed yet consequential genre—has received limited critical scrutiny. As a hybrid genre occupying the intersection of personal narrative and professional display, the scholar biography offers a unique site for examining how institutional norms, disciplinary ideologies, and cultural expectations converge in identity construction. Moreover, while existing studies have largely focused on monolingual or monocultural settings, comparative research across cultural and institutional contexts remains scarce. Addressing this gap could significantly enrich our understanding of how genre mediates the discursive construction of academic identities in a globalized knowledge economy.

## 3 Research design

### 3.1 Research questions

By comparing the personal bios of Chinese and English scholars in the domain of contrastive linguistics, this article aims to address the following research questions:

What rhetorical moves primarily constitute the bios of Chinese and English scholars, and what are their similarities and differences?
What types of identities are constructed in the bios of Chinese and English scholars, and how are these identities established?
What factors contribute to the differences in identity construction between the bios of Chinese and English scholars?

### 3.2 Research theories

**Figure 1:**
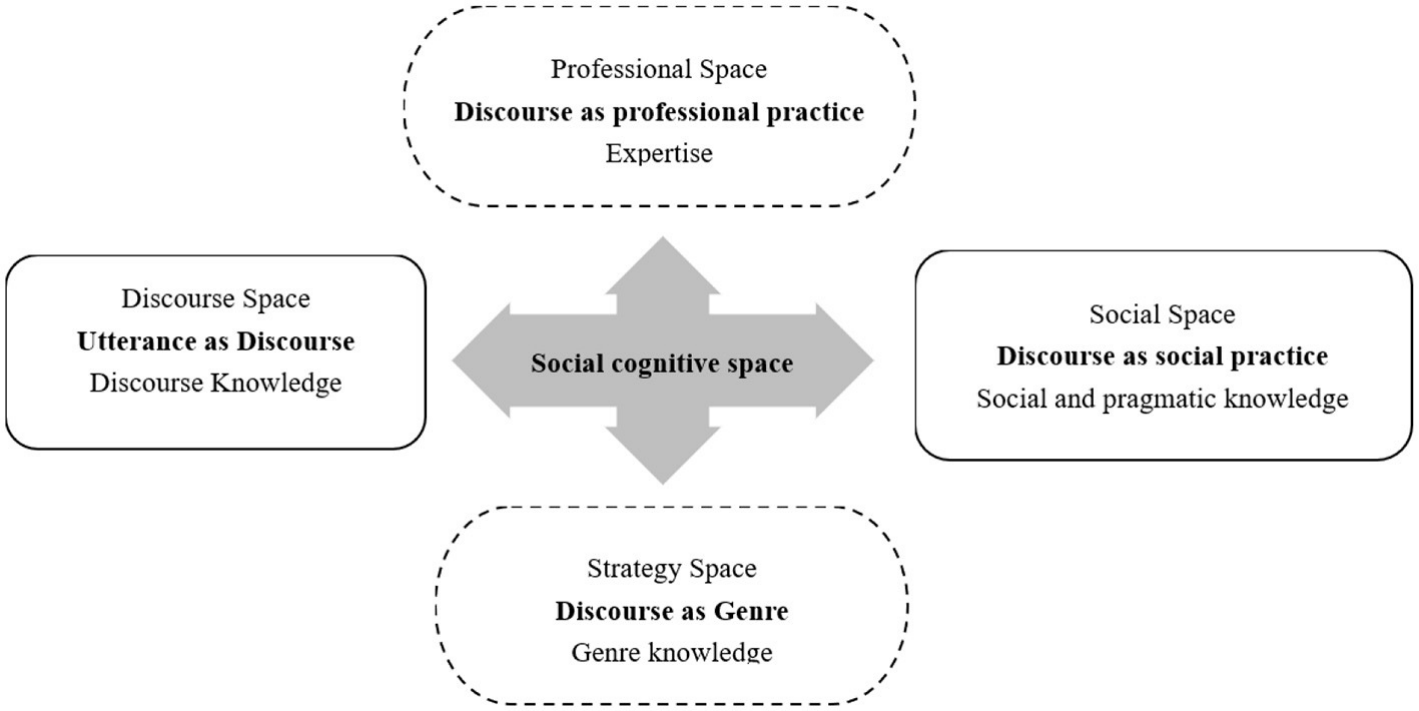
Discourse research perspective of CGA (Bhatia, 2008).

Among various theoretical models of genre analysis, critical genre analysis (CGA) extends the focus of traditional genre analysis from texts to their closely related contexts (Figure 1) and emphasizes multi-perspective and multidimensional analysis. This approach offers a contextually grounded analytical framework for examining discourse practices within specific communities (Ge and Wang, 2019). As perceived by Bhatia (2008), genre, a structural tool, plays a crucial role in discourse analysis, but its function should not be reduced to a fixed format. Instead, it should be viewed as a dynamic means of constructing and interpreting discourse within specific contexts. To fully understand the functions and meanings of professional discourse, it is essential to take into account both the formal features of language and the contextual environment where it is used (Jianguo and Congying, 2018).

In the present study, scholars' personal bios were categorized as an introductory genre of self-narrative (Hyland, 2018). First, the rhetorical move structures and identity types in the bios of Chinese and English scholars were identified by collecting, coding and statistically analyzing the corpus. Second, the lexical features in these bios were examined from the perspectives of professional expertise and discourse strategies, and their relationship with identity construction was explored. Finally, scholars' professional practices within social spaces and the influence of socio-cultural contexts were deeply investigated through interviews and the analysis of relevant literature.

### 3.3 Data collection

The academic biographical genre is shaped by a constellation of factors, including disciplinary conventions (Polizzi et al., 2021), situational contexts (Marques et al., 2024), and authorship positionality (Rose and Mckinley, 2022). Among the diverse modalities through which academic identities are discursively constructed, personal homepages hosted by scholars' affiliated institutions constitute a salient site for the public articulation of professional selves. This study, situated within the domain of linguistics, draws upon two authoritative evaluative frameworks to identify representative subjects: the *2022 Global Scientific Influence Rankings* published by Stanford University (Ioannidis, 2022) and the *Ranking of the Most Influential Chinese Scholars in Philosophy and Social Sciences* (Chang'an University Chinese Humanities Social Sciences Evaluation Research Center, 2020).

The *Global Scientific Influence Rankings*, grounded in bibliometric data from the Scopus database, adopt a composite scoring mechanism based on six key indicators: (1) total citation count, (2) Hirsch H-index, (3) Schreiber Hm-index (adjusted for co-authorship), (4) citations of single-authored publications, (5) citations of single- or first-authored publications, and (6) citations of single-, first-, or last-authored publications. In parallel, the Chinese ranking, compiled from databases such as CNKI and SuperStar Discovery, utilizes CNKI's disciplinary taxonomy to generate subfield-specific lists, including one dedicated to linguistics.

For the purposes of corpus construction, the personal homepages of the top 50 linguistics scholars from each ranking were systematically retrieved and analyzed. Data collection concluded in March 2023 (see Table 1, which presents the number of characters for Chinese and words for English). Inclusion criteria were as follows: (1) all subjects must be identified with the field of linguistics as per the respective rankings; (2) only extended, cohesive textual narratives were selected, while résumé-like formats or bullet-point entries were excluded—if the latter were encountered, the next ranked scholar was included as a replacement; (3) English-language bios were limited to scholars affiliated with institutions outside mainland China, irrespective of their native language, which falls beyond the scope of this inquiry; (4) attention was restricted to biographical texts published on institutional platforms, with the aim of examining how localized academic cultures mediate biographical self-presentation across comparable disciplinary spaces.

**Table 1:** List of corpus data used in the research.

**Category**	**Chinese**			**English**			**Total**
	**Male**	**Female**	**Total**	**Male**	**Female**	**Total**	
Number	33	17	50	27	23	50	100
Characters/Words	19,204	6,243	25,447	5,625	5,717	11,342	36,789

Based on the initial corpus analysis, the researcher further conducted semi-structured interviews to deepen the qualitative inquiry. Informed by the preliminary textual findings and aligned with the research objectives, a purposive sampling strategy was adopted to select interview participants. Specifically, a sampling frame was constructed by identifying a pool of scholars whose demographic attributes (i.e., nationality and gender) corresponded with the contextual variables of interest in this study. From each nationality-gender combination, one individual was selected using a simple random sampling procedure, resulting in a preliminary list of ten potential participants. These 10 scholars were formally contacted via email, in which the researcher clearly communicated the purpose of the study, the semi-structured nature and estimated duration of the interview, measures for confidentiality and anonymization, researcher contact details, and the voluntary nature of participation. Upon receiving affirmative responses and explicit informed consent, a total of five scholars agreed to participate in the interviews. Individual, in-depth semi-structured interviews were subsequently conducted with each of the five consenting participants. All interviews were scheduled at times and on platforms (including virtual meetings) convenient for the participants, with informed consent reconfirmed immediately prior to the sessions. Where permitted, audio recordings were made.

The process of data collection and analysis was iterative: transcription and coding were initiated promptly after each interview. The principle of “thematic saturation” was employed to determine the endpoint of data collection. After the fourth interview, no novel themes, patterns, or perspectives relevant to the research questions emerged, and sufficient depth and variation had been achieved in the interpretation of the existing data. Nevertheless, in adherence to prior commitments and to ensure the stability of saturation, the fifth interview was carried out as planned. Analysis of the fifth dataset confirmed the saturation point, as no substantially new or divergent insights were generated. In total, the interview data comprised 28,512 tokens of Chinese text and 18,104 words of English text. The interviews were designed to elicit participants' reflections on the rhetorical and contextual considerations involved in composing their personal bios. These insights offered valuable supplementary perspectives and enabled triangulation with the corpus-based findings. Details regarding the interview participants and procedures are summarized in Table 2.

**Table 2:** Information of respondents.

**Respondent**	**Language**	**Gender**	**Employment**	**Research area**	**Time**
A	Chinese	Male	Chinese University	Pragmatics and discourse analysis	32 min
B	Chinese	Male	Chinese University	Sociolinguistics and applied linguistics	26 min
C	Chinese	Female	Chinese University	Teacher education and language policy	38 min
D	English	Male	British University	Academic discourse analysis	30 min
E	English	Female	American University	Sociolinguistics	25 min
F	English	Female	British University	Educational linguistics	29 min

### 3.4 Data analysis

In this study, textual analysis was employed as a qualitative method. The coding framework for identifying rhetorical moves in the biographies was developed collaboratively by the author and a student with relevant academic training. Both the author and the student engaged in a detailed, iterative process to familiarize themselves with the coding principles, which were then applied to the selected biographies. The initial coding was followed by regular discussions to refine and validate the categories. To ensure inter-coder reliability, the initial coding was statistically tested using Cohen's Kappa. The calculated Kappa value was 0.85, indicating substantial agreement between the two coders. In cases of discrepancies, further discussion and clarification were carried out to achieve consensus. Any remaining disagreements were resolved through consultation with a third-party expert in discourse analysis.

The analysis focused on the identification and classification of rhetorical moves in the biographies. Each rhetorical move was described and interpreted to highlight genre-specific features related to identity construction and self-presentation. The AntConc tool was utilized to support the analysis by quantitatively identifying high-frequency terms, referential expressions, and transitivity patterns across the dataset. These linguistic features were compared across both Chinese and English biographies to examine the discourse practices in different sociocultural contexts.

To supplement the textual analysis, semi-structured interviews were conducted with scholars from both Chinese and English-speaking academic environments. The selection of interviewees was conducted in a scientifically rigorous and methodologically sound manner. Scholars were carefully chosen based on their academic backgrounds, expertise in relevant fields, and their experience with academic identity construction. A purposive sampling strategy was employed to ensure that participants could provide rich, relevant insights into the social factors influencing academic identity in scholar biographies. Additionally, the selection process was balanced to include scholars from diverse disciplines and academic stages, providing a broad range of perspectives. The interviews aimed to explore the social factors influencing the construction of academic identity in scholar biographies. These interviews were recorded, transcribed, and analyzed qualitatively to provide further insight into the discourse practices identified in the text analysis.

## 4 Results and discussion

### 4.1 Move structures in the introduction of Chinese and English scholars

As shown in Table 3, academic bios across both Chinese and English contexts generally follow a shared rhetorical structure, encompassing seven major moves: professional positions, research areas, educational background, publications, personal achievements, community service, and personal information. A chi-square test of independence revealed a statistically significant difference in the overall distribution of these rhetorical moves between the two corpora, χ^2^ (6, *N* = 694) = 27.33, *p* < 0.001, indicating language-specific tendencies in rhetorical construction.

**Table 3:** Percentage of each move in the biography of Chinese and English scholars.

**Move**	**Chinese**		**English**	
	**Plot**	**Percentage**	**Plot**	**Percentage**
Occupation	96	22.92%	70	25.46%
Research	46	10.98%	67	24.34%
Education	51	12.18%	25	9.10%
Publication	90	21.48%	42	15.28%
Achievement	58	13.84%	28	10.19%
Service	48	11.46%	22	8.00%
Personal profile	30	7.16%	21	7.64%
Total	419	100%	275	100%

Further item-wise analyses using two-proportion z-tests identified significant differences in the frequency of specific moves. Notably, the “Research” move appeared significantly more frequently in English-language bios (z = −4.67, *p* < 0.001), whereas the “Publication” move was more prevalent in Chinese-language bios (*z* = 2.04, *p* = 0.042). No statistically significant differences were observed for the other moves (all *p* > 0.05), including “Occupation,” which remained the most frequently occurring component across both corpora.

These findings suggest that while the macro-structural organization of academic bios remains largely consistent across linguistic contexts, scholars from different cultural backgrounds exhibit distinct rhetorical preferences in how they foreground their academic identity. Chinese scholars tend to emphasize professional titles, publication records, and academic achievements, reflecting a product-oriented representation of scholarly capital. In contrast, English-speaking scholars are more likely to highlight research areas, aligning with a discourse that foregrounds thematic expertise and intellectual positioning. For example:

(1) … *examines the historical and contemporary manifestation of raciolinguistic ideologies framing the language practices of racialized communities as inherently deficient and in need of remediation. He does this by undertaking raciolinguistic genealogies that situate the emergence of these raciolinguistic ideologies within European colonialism and traces the durability of these colonial logics across time and into the present. He has adopted this genealogical approach to reveal the ways that these colonial logics have historically informed and continue to inform United States (US) language education policies and practices as well as the ideological assumptions that have historically shaped and continue to shape the field of educational linguistics* … (2303E112)

(2) ……主要学术研究领域为语言学理论、现代汉语、心理语言学和语言规划……(2303C003)

… *Main academic research areas are linguistic theory, modern Chinese, psycholinguistics, and language planning* … (literal translation from 2303C003)

The two cases above illustrate that both Chinese and English scholars incorporate their research areas into their personal bios. In the bios of English scholars, however, this rhetorical move occupies a more significant portion. It often includes detailed accounts of their past research experiences and how these experiences have shaped their current research interests. This approach provides readers with a better understanding of their academic trajectories and research motivations while embodying the knowledge and skills they have accumulated in their specific fields.

Thus, it can be concluded that the types of rhetorical moves in the bios of Chinese and English scholars demonstrate a high degree of similarity, with minimal structural differences. This observation is in line with the framework of Hyland (2018). Nonetheless, socio-cultural contexts play a critical role in shaping the bios of scholars and influence the sequence of rhetorical moves and the level of detail provided in their content.

### 4.2 Discourse practices of identity construction in the biography of Chinese and English scholars

#### 4.2.1 Individual identity

Language acts as both a means and an institution of social construction, and functions as a fundamental tool for individuals to establish social identities (Ronghui, 2017). Naming strategies as a major form of identity recognition achieve this through referential or nominative expressions (Yongmei and Yanbin, 2013). Unsurprisingly, the bios of Chinese and English scholars in the collected corpus typically begin with scholars' names serving as a clear marker of their identity. Additionally, in English bios, as illustrated in Example (3), an adverbial clause introduced by ^*^after^*^ incorporates scholars' names into the text while describing their life and educational experiences. This approach enhances the coherence and readability of the text, while also making it easier for readers to understand the identity and characteristics of scholars:

(3) …* After studying German language and literature at the University of Paris-Sorbonne in the 1950s, Professor Kramsch emigrated to the US …* (2303E203)

In Example (4), the teaching experience is directly described from a first-person perspective. An individual identity that is both a teacher and a learner is constructed, which brings the author closer to readers.

(4) …* I first taught English as a foreign language in Malta in 1988, then graduated from the University of Malta in 1990 and began teaching English in a state secondary school also in Malta …* (2303E209)

Among the top 20 most frequent words, both Chinese and English academic profiles predominantly use nominalizations or verbal noun constructions, which help rationalize the organization of discourse and increase the coherence of structural layout in terms of prosody (Zhenhua and Chunxu, 2016). However, personal pronouns such as “she,” “he,” “I,” “her,” and “his” frequently appear in addition to these common features in English academic profiles, while self-referential language is notably absent in Chinese profiles. This observation is aligned with the findings of Na and Zhongqing (2013) in their study on personal pronouns in Chinese and English academic articles.

Apart from their referential function, personal pronouns also carry the nuanced functions of power, position, and politeness (Liu, 2024). They can convey an author's attitude toward the audience and thus contribute to analyzing the specific relationship between the author and readers. In English academic profiles, the use of first- (e.g., “I”) and third-person pronouns (e.g., “he” and “she”) expresses the presence and engagement of authors, fosters a closer connection with readers and encourages more active participation in text interpretation (Hyland, 2011). In contrast, Chinese academic profiles generally employ constructions without subjects, obscure the presence of writers, and underscore the informational content of discourse. By omitting personal pronouns, the focus is shifted to content, which thus highlights the authority of discourse and its subjects.

#### 4.2.2 Institutional identity

Burkitt (2008) argued that individuals tend to be categorized into specific groups when narrating their experiences, and the characteristics or traits associated with that identity are projected. In the context of contemporary higher education, marketization has become a trend in the development of universities (Fairclough, 1993). As a result, scholars commonly mention their current affiliation in personal profiles whether in Chinese or English, which is conducive to establishing their professional institutional identity and highlighting their affiliations and organizational contexts. For instance, Cases (5) and (6) explicitly state the affiliated institutions, academic titles and positions of individuals:

(5) ……北京外国语大学教授、博士生导师, 中国外语与教育研究中心专职研究员；北京外国语大学许国璋语言高等研究院院长；国家教材委员会外语学科专家委员会主任；中国英汉语比较研究会副会长；亚洲英语教学研究会副会长……(2303C212)

…* professor and doctoral supervisor at Beijing Foreign Studies University and full-time researcher at the China Foreign Language and Education Research Center; Dean of the Xu Guozhang Language Institute of Beijing Foreign Studies University; Director of the Foreign Language Subject Expert Committee of the National Textbook Committee; Vice President of the Chinese Association for Comparative Studies of English and Chinese; Vice President of the Asian English Teaching Research Association …* (literal translation from 2303C212).

(6) …* Paul Nation is an emeritus professor in Applied Linguistics at the School of Linguistics and Applied Language Studies (LALS) at Victoria University of Wellington, New Zealand …* (2303E108)

According to the theory of transitivity proposed by Halliday and Matthiessen, 2013, relational processes can be specifically classified into two types: “attributive” and “identifying”. In the process of introducing themselves, scholars usually establish connections between themselves and their peers and link these associations to broader socio-cultural contexts (Benwell and Stokoe, 2006). By employing “attributive relations,” scholars can express their membership in a particular community or their affiliation with an organization or discipline. Meanwhile, “identifying relations” are utilized to describe the relationship between scholars and a specific academic community, and depict the identity of scholars and their connection to the academic community in their fields. This is beneficial to reinforcing the institutional identity of scholars within a particular domain.

#### 4.2.3 Professional identity

As a rule, scholars use certain linguistic markers in their profiles to directly or indirectly demonstrate their professional identity (Guorong, 2019). These linguistic markers may contain information about scholars' research fields, research focus, achievements, academic titles, academic affiliations and membership in academic organizations. Moreover, the discourse of such profiles typically employs formal academic language and discourse styles, which are also considered as a way to exhibit their professional identity. For example, in the excerpt from Example (7), the research direction of scholars is directly introduced, which highlights the authors' extensive expertise and experience across multiple fields. Information such as participation in research projects, the approval of grants, and the publication of monographs and academic papers directly validate scholars' achievements in their areas of focus. More than that, the honors and titles scholars have received indirectly affirm the societal recognition of their professional contributions.

(7) ……主要从事认知语言学、语义学、语用学、语言与文化等方面的教学与研究工作。先后主持国家社会科学基金项目、世界自然基金会项目、湖南省社会科学基金项目、湖南省教育厅科研项目、湖南省社科联重点项目等。出版《英语常用词语辨析》、《英语词汇通》、《性别语言文化与语用研究》、《当代湖湘语言学者与外国语言学》等著作5部, 合著3部, 在国内重要学术刊物上发表论文50余篇……(2303C215)

…* mainly engaged in teaching and research in cognitive linguistics, semantics, pragmatics, language and culture, etc. He has successively presided over the projects of the National Social Science Foundation, the World Wildlife Fund and the Hunan Provincial Social Science Foundation, the scientific research projects of the Hunan Provincial Department of Education, the key projects of the Hunan Provincial Federation of Social Science, etc. He has also published five books including Analysis of Commonly Used English Words, English Vocabulary, Research on Gender Language Culture and Pragmatics, Contemporary Hunan Linguistic Scholars and Foreign Linguistics, and co-authored three books. These books are listed in important domestic academic journals. Not only that, he has published more than 50 papers …* (literal translation from 2303C215)

Material processes refer to the actions or activities involved in doing something. In academic profiles, material processes are used to describe the academic achievements of scholars and play a significant role in highlighting their “scholar identity.” By introducing the activities scholars have undertaken in the field, such as research projects, published papers and educational responsibilities, scholars can demonstrate their academic accomplishments and professional competence. This provides readers with a basis for evaluating the professional identity of scholars.

#### 4.2.4 Social identity

Social identity refers to the identity assumed by an individual and recognized by others within a social context. Individuals entering a social situation normally possess multiple social identities, which together form a composite of various roles (Xinren, 2018). In the discourse of academic profiles, some scholars also introduce their social identities apart from presenting information related to personal, institutional and professional identities. Below are Examples (8) and (9):

(8) ……曾任国家语言文字工作委员会副主任、教育部语言文字信息管理司司长、教育部语言文字应用研究所所长、《语言文字应用》杂志主编、中国社会科学院研究生院语言文字应用系主任、华中师范大学副校长……(2303C121)

…* has served as deputy director of the National Language and Writing Committee, director of the Language and Writing Information Management Department of the Ministry of Education (MOE), the Language and Writing Application Research Institute of the MOE, and the Language and Writing Application Department of the Graduate School of the Chinese Academy of Social Sciences, editor-in-chief of the Language and Writing Application magazine, and vice president of Central China Normal University…*(literal translation from 2303C121)

(9) …* He is the editor of the Bloomsbury Discourse Series and Routledge Innovations and Challenges in Applied Linguistics, founding co-editor of the Journal of English for Academic Purposes and co-editor of Applied Linguistics …* (2303E018)

Both the above-mentioned examples highlight the work identity of scholars outside of their research fields, and showcase the different roles and responsibilities they assume within society. This illustrates how the multiple social roles of an individual are interwoven and how they affect both their professional and social lives. This also confirms that identity is fluid, characterized by diversity and complexity, and likely to shift with changes in personal experiences, career development and social contexts.

Thus, the analysis and discussion above clearly show that academic profiles, a genre of discourse within a specific context, may be brief but contain rich information. Individuals employ discourse strategies in particular contexts and for specific needs to realize distinct communicative functions (Yuxin, 2016; Xinren, 2018). By selecting appropriate words, structures and expressions, scholars can shape their academic identity, and emphasize their expertise, research fields, achievements and social roles. This represents a strategy through which individuals adjust their identity expression according to their needs.

### 4.3 Socio-cultural factors of identity construction in the biography of Chinese and English scholars

#### 4.3.1 “A fixed template into which content can simply be inserted”

Different genres in different fields and occasions have an impact on the writing of scholar profiles. One interviewee said frankly during an interview:

“说实在的个人简介我还真没有那么细心去写, 在大家的心中, 应该就是一个模板式的内容写一下自己的相关信息。”(20240113C1)

“*… To be honest, I didn't write my profile carefully. In everyone's mind, it should be a template to write down relevant information about myself …”* (literal translation from 20240113C1)

It is evident that, in this institutionalized environment, self-introduction has turned into procedural discourse, where scholars can present themselves in a structured and professional manner. In this genre, how scholars can introduce themselves and the content needing to be included have already established implicit norms.

Another interviewee also mentioned that the identity presented in a self-introduction is a specific performative act and involves choices of language, expression and presentation. In academic or other professional contexts, authors can design their self-introductions in light of their intentions and the audience's expectations, to achieve a particular self-presentation effect:

“*… Identity is a performance and therefore can be changed. It's a performance reinforced over time. Hence, it becomes a habit. It is a disposition to behave in a certain way and make particular language choices. For this reason, we will continue to use language in that way if we are using language in a certain way and getting good feedback that we are being accepted as the kind of person we want to be seen as. This encourages certain identities.”* (20240109E1)

Academic profiles are a specialized genre within a specific field. They mirror certain consensus and norms within the academic community during the content-filling process, while also providing scholars with an effective means to convey personal information. The academic community has a relatively consistent understanding of the basic information that should be included in personal profiles, such as name, academic background and research focus. This consensus helps ensure that readers can quickly grasp the essential information of scholars without needing to search for key details. Furthermore, academic profiles as a professional genre typically require scholars to present their academic background, research achievements and scholarly contributions comprehensively within a limited space. The use of a template approach enables scholars to organize and present this information more effectively, which makes it easier for others to understand and evaluate.

Although the process of filling out an academic profile may seem mechanical, the individual differences and uniqueness embedded within it should not be neglected. Even when following a standardized template, each scholar still possesses unique academic experiences, research interests and educational background. In the process of filling in the template, scholars must skillfully highlight their personality and distinctive features to showcase their unique value within the structured format Likewise, one interviewee pointed out:

“……因为影响学者简介撰写的因素变量有很多, 很多时候可能会把它当作一个模板套路直接使用, 但是在使用的背后也会涉及到潜意识里想呈现的‘自我'的那一面……”(20240109C1)

“*… Because a number of factors and variables affect the writing of academic profiles, it is often used as a template. Behind the use of it, however, the side of the “self” that you want to present subconsciously is also involved …”* (literal translation from 20240109C1)

As a “procedural” genre, academic profiles are therefore not only a tool for conveying information but also an opportunity for scholars to present themselves on the academic stage. In the process of organizing and composing information, it is vital to skillfully balance norms and individuality, which ensures that academic profiles adhere to consensus principles while highlighting personal characteristics. This makes academic profiles an indispensable element of scholarly communication.

#### 4.3.2 “Very interesting power culture behind it”

As a social symbol, language has a quite obvious hierarchy. The order and length of the content presented have profound social implications, especially in the practice of fixed professional genres. This was mentioned by one interviewee during the interview:

“……简介作为一种模板, 他往往是有套路的。但是每个人在沿用这种模板的时候, 可能不同的人选择凸显的内容就不一样, 有的会把自己的求学经历放在特别凸显的位置, 而有的人直接写的是自己的工作单位, 背后所蕴含的权势文化是需要认真考虑的……” (20231019A1)

…* As a template, the introduction often follows a routine. However, different people may choose to highlight different things when using this template. Some people will put their education experience in a particularly prominent position, while others will directly write about their work units. The power culture behind it needs to be carefully considered …* (literally translated from 20231019A1)

Discourse functions foreground or background certain identities through the specific ways of structuring texts, while the sequence in which content is presented reflects the weighting and prioritization of information (Haiying, 2020). In acknowledging the template of the profile genre, interviewees further pointed out that the sequence where content is presented embodies a power dynamic worth attention. In fixed professional genre practices, the presentation order of content is usually not random but influenced by specific norms and traditions, and reflects the power structures and socio-cultural contexts within the academic community. Through the arrangement of content sequence, certain information may be emphasized, whereas other information is marginalized, with this power dynamic evident in the process of determining which information is considered more important and valued.

Another interviewee also noted, “We have to write in a way that shows positioning.” Scholars typically compose their personal profiles in a manner that showcases their identity and status. Explicit expressions may highlight their academic achievements, research focuses, teaching experience and other aspects to emphasize their positions within the academic community. Implicit expressions, on the other hand, maybe conveyed through language styles, word choices and modes of expression, communicate the professionalism and confidence of scholars, and thereby construct their status within the academic community.

…* Okay, it isn't just doing something or using language in identity but doing something with other people. It depends on who we are talking to, who we are writing for and what they expect to find in that text. I write research articles because I want to reach an audience of academics and teachers. I don't write poems or rap songs as I'm not writing for those audiences. Thus, we have to write in a way that shows positioning …* (20240109E5)

The process of identity construction in this context is dynamic and involves individuals' adjustment of behavior and beliefs in different cultural environments. In writing, individuals may choose to emphasize content that aligns with specific values. Hence, they can adapt to the expectations of different cultures, disciplinary fields and professional communities for better integrating into and gaining recognition within the academic community and establishing connections with readers. This also manifests the interactive relationship between scholars' institutional identity within the academic world and societal values as well as their positioning and self-recognition within this social system. The ability to understand and respond to the expectations within different socio-cultural contexts facilitates the participation of scholars in international academic exchanges, expands collaborative relationships and helps them gain recognition in a diverse academic environment.

#### 4.3.3 “Too competitive academic environment”

Peer competition has a profound impact on academic profiles, and requires scholars to pay more attention to their competitiveness and brand image in the academic market and demonstrate their personal value through multiple communication channels. This makes academic profiles not only a form of academic communication but also one of the key factors for success in the academic market:

…* I imagine that the bios of Chinese scholars are longer and more complex because they may try harder to present themselves more academically and attempt to distinguish themselves from thousands of Chinese professors due to greater competition ..*. (20240120E1)

As the interviewee mentioned, personal profiles have become a key tool for scholars to demonstrate their unique value and distinguish themselves from others in a fiercely competitive environment with the marketization of academic research and education. Scholars shall highlight their academic achievements, research contributions and professional expertise through personal profiles to stand out in the academic market:

…* I think we are generally trying to represent ourselves as someone to be accepted by a group ... Therefore, by looking at language, we are writing to be part of the group to gain the acceptance and prestige of that group and the advantages that give us …* (20240120E3)

The influence of marketization is not limited to the individual level but also exerts a far-reaching on the identity construction within the same community group. When pursuing the construction of their individual identities, scholars must also take their positions and relational networks within the academic community into consideration. The construction of identity is no longer merely an individual matter but involves the collaboration and interaction of the entire academic community. When showcasing their personal brands, scholars must also maintain alignment with the values and shared goals of the community to preserve cohesion and cooperation within the community. Therefore, scholars are not only constructors of their individual and institutional identities but also shapers of their professional and social identities in a marketized academic environment. They must find a balance between individual differences and community identity to better adapt to and shape the evolving academic ecosystem.

## 5 Conclusions

This study conducted a comparative analysis of Chinese and English academic profiles in the field of linguistics, employing Critical Genre Analysis (CGA) within the theoretical framework of social constructivism of identity. By examining the discourse functions embedded in these scholar biographies, the research revealed how identity is strategically constructed and negotiated through linguistic choices, structural patterns, and rhetorical strategies. It highlighted the nuanced ways in which scholars from different cultural backgrounds navigate institutional expectations, disciplinary norms, and personal identity expression, thereby shedding light on the dynamic interplay between discourse and identity construction.

The findings demonstrate that academic profiles are far more than static self-introductions; they function as purposeful, socially situated texts that reflect broader socio-cultural, professional, and ideological forces. Through the identification of common step structures and identity types, as well as the analysis of interview data, this study uncovered the contextual factors shaping scholars' self-presentation practices. These insights enrich the current understanding of how professional identities are discursively constructed across cultural contexts, and they underscore the role of genre in mediating personal voice, disciplinary alignment, and institutional legitimacy.

Nonetheless, this study has its limitations. To control for external variables and ensure consistency, the data were drawn solely from self-introduction texts on academic webpages within a single discipline—linguistics. While this focused scope allows for depth of analysis, it also limits the generalizability of the findings. Future research could broaden the investigation by exploring how the same scholars present their identities across multiple platforms (e.g., social media, conference bios, grant applications), or by comparing identity construction practices between novice and senior scholars, as well as across different disciplines and institutional types. In particular, cross-contextual and longitudinal studies may further illuminate how academic identities evolve over time and are shaped by shifting professional demands and socio-cultural dynamics.

In conclusion, this study contributes to the growing body of literature on academic discourse and identity by foregrounding the scholarly biography as a valuable site for exploring identity performance. It calls for increased scholarly attention to this often-overlooked genre and encourages further inquiry into the discursive, cultural, and institutional dimensions of academic self-representation.

## Data Availability

The raw data supporting the conclusions of this article will be made available by the authors, without undue reservation.
